# Investigating the Impact of the New York State Flavor Ban on e-Cigarette–Related Discussions on Twitter: Observational Study

**DOI:** 10.2196/34114

**Published:** 2022-07-08

**Authors:** Yankun Gao, Zidian Xie, Dongmei Li

**Affiliations:** 1 University of Rochester Medical Center Rochester, NY United States

**Keywords:** New York State flavor ban, e-cigarettes, twitter, topic modeling, sentiment analysis

## Abstract

**Background:**

On May 18, 2020, the New York State Department of Health implemented a statewide flavor ban to prohibit the sales of all flavored vapor products, except for tobacco or any other authorized flavor.

**Objective:**

This study aims to investigate the discussion changes in e-cigarette–related tweets over time with the implementation of the New York State flavor ban.

**Methods:**

Through the Twitter streaming application programming interface, 59,883 e-cigarette–related tweets were collected within the New York State from February 6, 2020, to May 17, 2020 (period 1, before the implementation of the flavor ban), May 18, 2020-June 30, 2020 (period 2, between the implementation of the flavor ban and the online sales ban), July 1, 2020-September 15, 2020 (period 3, the short term after the online sales ban), and September 16, 2020-November 30, 2020 (period 4, the long term after the online sales ban). Sentiment analysis and topic modeling were conducted to investigate the changes in public attitudes and discussions in e-cigarette–related tweets. The popularity of different e-cigarette flavor categories was compared before and after the implementation of the New York State flavor ban.

**Results:**

Our results showed that the proportion of e-cigarette–related tweets with negative sentiment significantly decreased (4305/13,246, 32.5% vs 3855/14,455, 26.67%, *P*<.001), and tweets with positive sentiment significantly increased (5246/13,246, 39.6% vs 7038/14,455, 48.69%, *P*<.001) in period 4 compared to period 3. “Teens and nicotine products” was the most frequently discussed e-cigarette–related topic in the negative tweets. In contrast, “nicotine products and quitting” was more prevalent in positive tweets. The proportion of tweets mentioning mint and menthol flavors significantly increased right after the flavor ban and decreased to lower levels over time. The proportions of fruit and sweet flavors were most frequently mentioned in period 1, decreased in period 2, and dominated again in period 4.

**Conclusions:**

The proportion of e-cigarette–related tweets with different attitudes and frequently discussed flavor categories changed over time after the implementation of the New York State ban of flavored vaping products. This change indicated a potential impact of the flavor ban on public discussions of flavored e-cigarettes.

## Introduction

Electronic cigarettes (e-cigarettes) are battery-powered devices that allow users to inhale substances by heating vaporized liquid, which usually contains nicotine, flavoring agents, propylene glycol/vegetable glycerin, and other additives [1]. In recent years, e-cigarette use has increased rapidly among youth and young adults in the United States. From 2014 to 2020, e-cigarettes were the most commonly used tobacco product among high school and middle school students [2]. The JUUL e-cigarette system’s compact design, high nicotine content, and myriad of flavor options allowed it to dominate the US e-cigarette market by 2018, and it has become increasingly popular among teens since mid-2015 [2,3]. Multiple studies have suggested that e-cigarette use is associated with adverse health consequences, such as respiratory disorders, mental health problems, cognitive issues, and cancers [4-9]. In addition, e-cigarette use is unlikely to help adult smokers quit smoking [10]. By contrast, young people who use e-cigarettes are more likely to initiate smoking than those who do not [11], and the nicotine in e-cigarettes can increase the risk of addiction to other substances [12].

Flavored tobacco products can hide the harsh taste of tobacco, and the various flavors available have become the most attractive feature of e-cigarettes for youth and young adults [13,14]. An estimated 72.2% of high school and 59.2% of middle school students reported using flavored e-cigarettes, with the most popular flavors being fruit, menthol or mint, and various sweets [2]. Compared to adults, youth were more likely to use multiple flavor categories, and the most reported combination was fruit and candy [15]. While many flavors contained in e-liquids are used as food additives and scents, there are concerns about their safety in the lung [16,17]. Studies have suggested that the flavoring chemicals in e-cigarettes can harm lung tissue by impairing the cilia function in the airway epithelium and imposing inflammatory and oxidative responses in lung cells [18-20]. The inhalation of cinnamaldehyde, a flavoring agent commonly used in e-cigarettes, may increase the risk of respiratory infections in e-cigarette users [21]. The presence of vanillin is related to higher toxicity values, and the concentration of vanillin and cinnamaldehyde has been correlated with toxicity [22]. In addition, the variety of e-cigarette flavors has rapidly grown. While 7764 unique e-cigarette flavors were reported on the internet in January 2014, there was a net increase of 242 new flavors monthly in the 17 months that followed [23].

Due to their potential adverse health consequences, various policies have been announced or implemented to protect young people from flavored e-cigarettes. On November 15, 2018, the US Food and Drug Administration (FDA) implemented age restrictions on the sale of flavored e-cigarettes in physical locations and heightened age verification procedures for online sales [24]. On June 25, 2019, San Francisco banned the sale and distribution of e-cigarettes in the city to keep them away from young people [25]. On September 17, 2019, New York State announced its first-in-the-nation policy on flavored e-cigarettes [26]. That same year, multiple states and counties passed similar bans on the sale of flavored vaping products [3,27-30]. On January 2, 2020, the FDA announced their flavor enforcement policy to restrict flavored vaping products other than tobacco and menthol flavors, which were less popular among teenagers, and implemented the policy on February 6, 2020 [31]. On May 18, 2020, the New York State Department of Health implemented a flavor ban to prohibit the sales of all flavored vapor products, except for tobacco flavor and any flavored product that received a premarket approval order from the FDA, and banned the online sale of any vapor products on July 1, 2020 [32,33].

In this study, we aim to investigate sentiment changes in e-cigarette–related tweets over time with the implementation of the New York State flavor ban. We used Twitter data for this research since global tweets are updated continuously, allowing us to track public opinion in real time, which traditional surveys cannot generally provide. Twitter is a popular social media platform in the United States, with many users being teenagers and young adults [34]. In addition, previous studies successfully used Twitter data to study public perceptions of e-cigarette and e-cigarette–related policies [35,36]. In this study, we compared the sentiment and topic changes in e-cigarette–related tweets over time. In addition, we aimed to examine the changes in e-cigarette flavors mentioned on Twitter with the implementation of the New York State flavor ban. The findings of this study can provide insights into the potential impact of the flavor ban, which can be helpful for other policies on the regulation of flavored e-cigarettes.

## Methods

### Data Collection

E-cigarette–related tweets were collected through the Twitter streaming application programming interface (API) using e-cigarette–related keywords, including “e-cig,” “e-cigs,” “ecig,” “ecigs,” “electroniccigarette,” “ecigarette,” “ecigarettes,” “vape,” “vapers,” “vaping,” “vapes,” “e-liquid,” “ejuice,” “eliquid,” “e-juice,” “vapercon,” “vapeon,” “vapefam,” “vapenation,” and “juul” [37-39]. Twitter data were collected during 4 time periods: February 6, 2020-May 17, 2020 (before the implementation of the New York State flavor ban), May 18, 2020-June 30, 2020 (between the implementation of the flavor ban and the online sales ban), July 1, 2020-September 15, 2020 (the short term after the online sales ban), and September 16, 2020-November 30, 2020 (the long term after the online sales ban). September 15, 2020, was the midpoint of the data collected after implementing the flavor ban and therefore used as the cutoff between periods 3 and 4. As a result, a total of 2,929,784 unique e-cigarette–related tweets were collected.

To remove e-cigarette promotion tweets, Twitter posts and IDs were filtered out using promotion-related keywords, including “dealer,” “deal,” “customer,” “promotion,” “promo,” “promos,” “discount,” “sale,” “free shipping,” “sell,” “$,” “%,” “dollar,” “offer,” “percent off,” “store,” “save,” “price,” and “wholesale” [40]. After the promotion filtering, the data set contained 2,298,791 unique e-cigarette–related tweets. To investigate e-cigarette–related tweets within the state of New York State, geolocation keywords that contained city names related to the state, such as “New York,” “NY,” “Syracuse,” “Buffalo,” and so forth were used to filter users' geolocations. As a result, 59,883 e-cigarette–related tweets within the state of New York were obtained, with period 1 having 24,976 unique tweets, period 2 having 7206 unique tweets, period 3 having 13,246 unique tweets, and period 4 having 14,455 unique tweets.

### Sentiment Analysis

The Valence Aware Dictionary and Sentiment Reasoner (VADER) was used as the sentiment analyzer to compute Twitter users’ attitudes [41]. A sentiment score was calculated for each tweet, ranging from −1.00 to +1.00. The attitudes of tweets were defined as positive if sentiment scores were in the range of +0.05 to +1.00, neutral if sentiment scores were in the range of −0.05 to +0.05, and negative if sentiment scores were in the range of −1.00 to −0.05. A score of −0.05 was included in the negative sentiment group and that of +0.05 was included in the positive sentiment group. To compare the sentiments between different periods, the number of tweets with different sentiments was normalized by the total number of tweets within each period. The 2-proportion *Z* test was used to compare the proportions of positive, neutral, and negative tweets between the different periods [42].

### Topic Modeling

Topic modeling, specifically the latent Dirichlet allocation (LDA) model, was conducted to analyze the Twitter text content and determine the most frequently discussed topics. LDA is a generative model for text modeling, in which each word is assigned to a topic, and topics are generated with keywords and their corresponding weights [43]. LDA modeling was applied to tweets with different attitudes in the 4 periods. First, uppercase characters were converted to lowercase, and all punctuation, stop words, and white spaces were removed. Then, the Python library Genism was used to identify some frequent bigrams and trigrams [44]. Words were then lemmatized using spaCy to make all tenses present, and only nouns, verbs, adjectives, and adverbs were kept [44]. After all the data cleaning processing procedures, coherence scores were calculated, and the maximum coherence score was used to determine the number of topics [45]. Finally, the keywords and the percentage distribution of each topic were visualized with the pyLDAvis package [46].

### Flavor Frequency

Further filtering was conducted using 1198 e-liquid flavor names from 129 e-liquid brands to identify tweets mentioning flavors [40]. As a result, 3714 tweets were collected in period 1, 1764 in period 2, 2027 in period 3, and 3544 in period 4. The e-cigarette flavors were grouped into 8 categories, including fruit, sweets, beverage, mint, menthol, tobacco, mixed, and other [40]. The proportions of different flavor categories were calculated by dividing the number of tweets mentioning each category by the total number of e-cigarette–related tweets in the same period. The proportions of each flavor category were compared between different periods by using the 2-proportion *Z* test with a significance level of 5%.

### Ethics Approval

Only publicly available tweets were used for this study. There was no identifying information on Twitter users in this study. In addition, this study was reviewed and approved by the Office for Human Subject Protection Research Subjects Review at the University of Rochester (study ID STUDY00006570).

## Results

### Public Attitudes in e-Cigarette–Related Tweets in New York

To investigate whether the New York flavor ban could potentially affect public sentiments in e-cigarette–related tweets, the proportions of positive, neutral, and negative e-cigarette–related tweets before and after the flavor ban were compared at different periods using 2-sided two proportion *Z* tests (Multimedia Appendix 1). When comparing period 2 (May 18, 2020-June 30, 2020) to period 1 (February 6, 2020-May 17, 2020), we found no significant difference in the proportions for all attitudes (2738/7206, 38% vs 9342/24,976, 37.4% for positive, *P*=.35; 2054/7206, 28.5% vs 7381/24,976, 29.55% for neutral, *P*=.07; and 2414/7206, 33.5% vs 8253/24,976, 33.04% for negative, *P*=.43). From period 2 (May 18, 2020-June 30, 2020) to period 3 (July 1, 2020-September 15, 2020), the proportion of positive discussions significantly increased (2738/7206, 38% vs 5246/13,246, 39.6%, *P*=.025), while the proportions of neutral and negative tweets did not show significant changes (2054/7206, 28.5% vs 3694/13,246, 27.89%, *P*=.36 and 2414/7206, 33.5% vs 4305/13,246, 32.5%, *P*=.15, respectively). However, the proportion of both neutral (3694/13,246, 27.89% vs 3562/14,455, 24.64%, *P*<.001) and negative (4305/13,246, 32.5% vs 3855/14,455, 26.67%, *P*<.001) tweets significantly decreased, and the proportion of positive tweets (5246/13,246, 39.6% vs 7038/14,455, 48.69%, *P*<.001) significantly increased in period 4 (September 16, 2020-November 30, 2020) compared to period 3 (July 1, 2020-September 15, 2020).

### Topics Discussed in e-Cigarette–Related Tweets in Different Periods

To determine if there was any change in e-cigarette–related topics discussed before and after the implementation of the New York State flavor ban, we applied the LDA topic modeling to e-cigarette–related Twitter posts from New York State. The popular topics discussed in the positive and negative tweets at different time periods are summarized in Table 1. We noticed that the popular topics in tweets with a positive sentiment were similar in all 4 periods. The majority of tweets focused on topics related to “nicotine products (include vaping and smoking) and quitting.” Moreover, 2 other topics, “nicotine products and health” and “nicotine products and people behavior,” were also frequently discussed in the positive tweets. However, while the “nicotine products and quitting” and “nicotine products and people behavior” topics also appeared in the tweets with a negative sentiment, “teens and nicotine products” was the most frequently discussed topic in the negative tweets in periods 1, 2, and 4. In period 2, “mint flavor vape in New York” became one of the most popular topics in the negative tweets.

**Table 1:** Top topics related to e-cigarettes before and after the New York State flavor ban.

Periods	Positive sentiment			Negative sentiment	
	Topics (% of tokens)	Keywords	Topics (% of tokens)		Keywords
February 6, 2020, to May 17, 2020^a^ (24,976 posts)	Nicotine products and quitting (40.8)	vap, vape, nicotine, smoking, quit, cigarette, smoker, smoke, help, people, product, amp, tobacco, vaper, vaping	Teens and nicotine products (55.4)		vap, cigarette, nicotine, vape, amp, smoke, product, smoking, people, not, lung, quit, tobacco, teen, vaper
	Nicotine products and people behavior (36.1)	vape, juul, vap, get, go, smoke, good, not, be, make, pod, love, want, hit, do	Nicotine products and quitting (35.2)		juul, vape, get, go, vap, smoke, stop, pod, lose, not, be, fuck, shift, make, hit
May 18,2020, to June 30, 2020^b^ (7206 posts)	Nicotine products and quitting (47.4)	vape, vap, juul, smoke, get, not, good, nicotine, go, be, people, quit, do, cigarette, smoking	nicotine products and quitting (54.9)		vap, vape, nicotine, smoke, amp, cigarette, tobacco, smoking, not, people, say, quit, year, product, vaper
	Nicotine products and health (26.9)	vap, smoker, vaper, smoke, nicotine, smoking, vape, product, quit, amp, help, tobacco, worldvapeday, health, study	mint flavor vape in New York (30.1)		vape, juul, fuck, pod, cana, anything, get, mint, new_York, month, people, get_rid, hit, police_force, like_two
July 1,2020, to September 15, 2020^c^ (13,246 posts)	Nicotine products and people behavior (42.6)	vape, juul, get, vap, smoke, good, be, hit, go, not, make, amp, know, day, pen	Teens and nicotine products (48.3)		vap, stop, nicotine, white_people, pray, whole_time, downfall, mom_asking, vape, cigarette, smoke, smoking, vaper, tobacco, amp, teen
	Nicotine products and quitting (41.8)	vap, nicotine, vape, smoking, smoke, cigarette, quit, tobacco, smoker, product, map, vaping, vaper, not, help	Nicotine products and addiction (45.4)		vape, juul, get, vap, fuck, addict, go, smoke, drink, not, be, s, shit, pod, mad
September 16, 2020, to November 30, 2020^d^ (14,455 posts)	Nicotine products and quitting (64%)	vape, vap, nicotine, smoke, juul, cigarette, good, get, smoking, quit, amp, smoker, people, be, go	Teens and nicotine products (54.2)		vap, vape, nicotine, smoke, not, smoking, teen, cigarette, quit, people, tobacco, do, amp, smoker, vaper
	Nicotine products and people behavior (26%)	vape, smoke, xbox, blow, series, not, say, can, believe, get, buy, bottom, feel_proud, twitter, created_post	Nicotine products and people behavior (36.3)		vape, juul, vap, go, get, fuck, pen, ita, shit, hit, be, lose, bad, take, dona

^a^Before implementation of the New York State flavor ban.

^b^Between the implementation of the New York State flavor ban and the online sales ban.

^c^Short term after the online sales ban.

^d^Long term after the online sales ban**.**

### e-Cigarette Flavors Mentioned on Twitter

To investigate the potential effects of the New York State flavor ban on e-cigarette flavors discussed on Twitter, we compared the proportions of the e-cigarette flavor categories over time (Multimedia Appendix 2). Compared to period 1, in period 2, the proportions of tweets mentioning mint (296/3714, 8% vs 321/1764, 18%, *P*<.001) and menthol (312/3714, 8.4% vs 340/1764, 19%, *P*<.001) flavors significantly increased, while the proportions of tweets mentioning fruit (1559/3714, 41% vs 674/1764, 38%, *P*<.05), sweet (829/3714, 22.3% vs 253/1764, 14%, *P*<.001), beverage (456/3714, 12.3% vs 103/1764, 6%, *P*<.001), and tobacco (164/3714, 4.4% vs 39/1764, 2%, *P*<.001) flavors significantly decreased. After the ban of the online sale of all vapor products, the proportion of tweets mentioning mint (321/1764, 18% vs 246/2027, 12%, *P*<.001) and menthol (340/1764, 19% vs 278/2027, 14%, *P*<.001) significantly decreased in period 3 compared to period 2 but was still significantly higher than that in period 1 (246/2027, 12% vs 296/3714, 8% and 278/2027, 14% vs 312/3714, 8.4%, respectively, *P*<.001 for both flavors). At the same time, the proportions of tweets mentioning sweets (253/1764, 14% vs 340/2,027, 17%, *P*<.05), beverage (103/1,764, 6% vs 216/2,027, 11%, *P*<.001), and tobacco (39/1764, 2% vs 80/2027, 4%, *P*<.001) flavors significantly increased in period 3 compared to period 2. In period 4, a few months after the implementation of the New York State flavor ban, the proportion of tweets mentioning mint (246/2027, 12% vs 118/3544, 3%, *P*<.001), menthol (278/2027, 14% vs 160/3544, 5%, *P*<.001), beverage (216/2027, 11% vs 160/3544, 5%, *P*<.001), and tobacco (80/2027, 4% vs 68/3544, 2%, *P*<.001) significantly decreased, while the proportion of tweets mentioning fruit (819/2027, 40% vs 2004/3544, 57%, *P*<.001) and sweet flavors (340/2027, 17% vs 976/3544, 28%, *P*<.001) significantly increased compared to period 3.

## Discussion

### Principal Findings

To reduce the use of flavored e-cigarettes by young people and limit the appeal of flavored e-cigarette products, New York State implemented a flavor ban on May 18, 2020, and an online sales ban on July 1, 2020 [32,47]. In this study, we showed that after the implementation of the flavor ban, the proportion of positive e-cigarette–related tweets in New York State significantly increased over time, while the proportion of tweets with neutral or negative sentiments significantly decreased. The main topics in positive tweets were nicotine products, quitting, and health in all 4 periods, while the negative tweets focused on teens and nicotine products. Among all the tweets mentioning flavors, the proportion of tweets mentioning mint or menthol flavors significantly increased right after the implementation of the New York flavor ban and then gradually decreased.

### Comparison With Prior Work

In this study, we showed that the most frequently mentioned flavors before implementing the New York State flavor ban were fruit (1559/3714, 42%) and sweets (829/3714, 22.3%), which was consistent with previous studies that fruit and sweet flavors are the most popular e-cigarette flavor categories [40]. Before the New York State flavor ban, the FDA implemented a flavor enforcement policy to restrict closed system devices containing flavored liquids other than tobacco and menthol flavors, which were less preferred by teenagers [31]. Different types of e-cigarettes (eg, disposable e-cigarettes [48]) might have partially contributed to the high proportions of fruit and sweet flavors discussed in period 1.

Right after the implementation of the New York State flavor ban, the percentage of tweets mentioning menthol increased to 19% (340/1764) in period 2, compared to 8.4% (312/3714) in period 1 (Multimedia Appendix 2). The ban limited the sales of menthol-flavored vapor products, which the FDA enforcement policy allowed. Therefore, many discussions around the newly banned menthol flavor appeared on Twitter in period 2, such as “I had a dream i went to the gas station and my menthol juul pods were back” and “They should bring back the Menthol juul pod so people can stay off of Newports cuz Newports kill the hood that's facts...” The proportion of menthol flavor–related discussion declined to 5% (160/3544) by period 4, which indicated the potentially reduced availability of menthol-flavored vapor products in New York State.

Topics related to nicotine products and quitting were the most prevalent in tweets with positive sentiments, such as “I’m so much happier ever since i decided to be a healthier person and quit vaping and smoking weed.” However, in negative tweets, the topic of teens and nicotine products was actively discussed, such as “All e-cigarette advertising is anti-smoking advertising. And smoking actually kills people. A LOT of people. Nicotine vaping does not. Anti-vaping advertising is well-funded and well-orchestrated. Their ads increase teen vaping and discourage smokers from quitting.” In period 4, the proportion of negative e-cigarette–related tweets significantly decreased (3855/14455, 26.67%) compared to period 3 (4305/13,246, 32.5%). These dramatic changes in the proportions of positive and negative tweets did not occur until period 4, a few months after the implementation of the flavor ban, which suggest an association between the policy and sentiment changes in e-cigarette–related tweets. “Teens and nicotine products” was a major topic discussed in both periods 3 and 4. The declined proportion indicated that public concerns about teen vaping might have reduced over time after the New York State flavor ban. 

The youth initiation of e-cigarette use was associated with flavored e-cigarette products, and fruit and sweet flavors are the most popular flavor categories among youth in the United States [15,40,49,50]. Mint flavor is one of the most often used JUUL e-cigarettes flavors in middle school and high school students [51]. However, after the sale of all unauthorized flavors was banned in New York State, there was a significantly increased proportion of mint flavor–related tweets (321/1764,18% in period 2 vs 296/3714, 8% in period 1), while the proportions of tweets mentioning fruit, sweet, and beverage flavors decreased. Consistent with this result, mint-related discussions became a major topic in the negative tweets in period 2. In this period, there was one popular tweet, “How did New York get rid of mint juul pods in like two months but can’t do anything about the exceedingly racist police force?” This tweet mentioned mint flavor and other political topics and has since been retweeted virally, which might explain why the mint flavor became prominent among all the banned flavors in period 2.

Tobacco flavor is less preferred by US youth [51]. The New York State flavor ban prohibited the sales of all flavors other than tobacco. Multiple studies show that e-cigarette consumers are willing to shift to different flavors when certain popular flavors are restricted [50,52]. However, we did not observe an increase in tobacco flavor–related discussions after the New York State flavor ban. In contrast, discussions about fruit and sweet flavors still dominated the tweets mentioning flavors. One reason might be that the amount of discussions on social media does not necessarily reflect the amount of e-cigarette use in real life. Another possible explanation might be that people turn to other sources to get the flavored e-cigarettes they like, such as buying from other states or the black market.

### Limitations

This study has several limitations. First, we collected tweets from New York State using the geographical location of users for our analysis. However, most Twitter users are not willing to share their locations in their tweets [53], which might have introduced some biases to our study. Second, we aimed to analyze the potential effects of the New York flavor ban on public attitudes and user behaviors. However, the FDA enforcement policy was implemented a few months before the ban. Therefore, the changes we observed in this study might have been a combination of the 2 policies other than the flavor ban alone. Moreover, e-cigarette bans were not new to the United States at the time of the New York State ban, and attitudes collected via Twitter may have been a reaction to this long-term trend rather than an acute event. Third, we investigated the changes in attitudes of e-cigarette–related tweets before and after the New York State flavor ban, which only reflects general discussion rather than self-reports of e-cigarette usage. Therefore, whether people quit vaping or switched to other available nicotine products after the flavor ban remains unknown and will be further pursued in future studies. Fourth, our study only showed the association between flavor ban and the attitude changes in e-cigarette–related tweets, which cannot determine the causal effects of the New York flavor ban on public attitudes. Finally, there were only 5 months of data collected after the implementation of the flavor ban. As a result, public discussions of e-cigarettes in the longer term remain unknown.

### Conclusions

Using Twitter data, this study showed the changes in public attitudes in e-cigarette–related discussions and changes in the proportion of e-cigarette flavor categories mentioned before and after the New York State flavor ban. Our results indicated that the public might have less concerns about teen vaping after the implementation of the flavor ban. In addition, our results showed that while the mentions of some banned flavors (eg, mint and menthol) temporally increased right after the flavor ban, the most popular flavors (eg, fruit and sweets) dominated discussions again over time. These results indicated that stricter nationwide policies are required to prohibit flavored nicotine products. The findings of this study provide some preliminary evidence of public responses to the New York State flavor ban as well as valuable insights for policy makers to further regulate flavored nicotine products.

## Appendix

Multimedia Appendix 1 Sentiment changes in e-cigarette–related tweets with the implementation of the New York State flavor ban. * indicates *P*<.05, ** indicates *P*<.01. February 6, 2020-May 17, 2020, before implementation of the flavor ban; May 18, 2020-June 30, 2020, between the implementation of the flavor ban and the online sales ban; July 1, 2020-September 15, 2020, the short term after the online sales ban; September 16, 2020-November 30, 2020, the long term after the online sales ban.

Multimedia Appendix 2 Proportions of tweets mentioning e-cigarette flavor categories over time before and after the New York State flavor ban. February 6, 2020-May 17, 2020, before implementation of the flavor ban; May 18, 2020-June 30, 2020, between the implementation of the flavor ban and the online sales ban; July 1, 2020-September 15, 2020, the short term after the online sales ban; September 16, 2020-November 30, 2020, the long term after the online sale ban.

## Abbreviations

API: application programming interface
FDA: Food and Drug Administration
NIH: National Institutes of Health
LDA: latent Dirichlet allocation
VADER: Valence Aware Dictionary and Sentiment Reasoner

## Data Availability

The data and scripts used for analysis and creating figures are available upon request from the corresponding author.
