# Supplementary figures and images for: Investigating the Impact of the New York State Flavor Ban on e-Cigarette–Related Discussions on Twitter: Observational Study
Source: JMIR Public Health Surveill. 2022 Jul 8;8(7):e34114. doi: 10.2196/34114 (PMC9308079; doi:10.2196/34114)

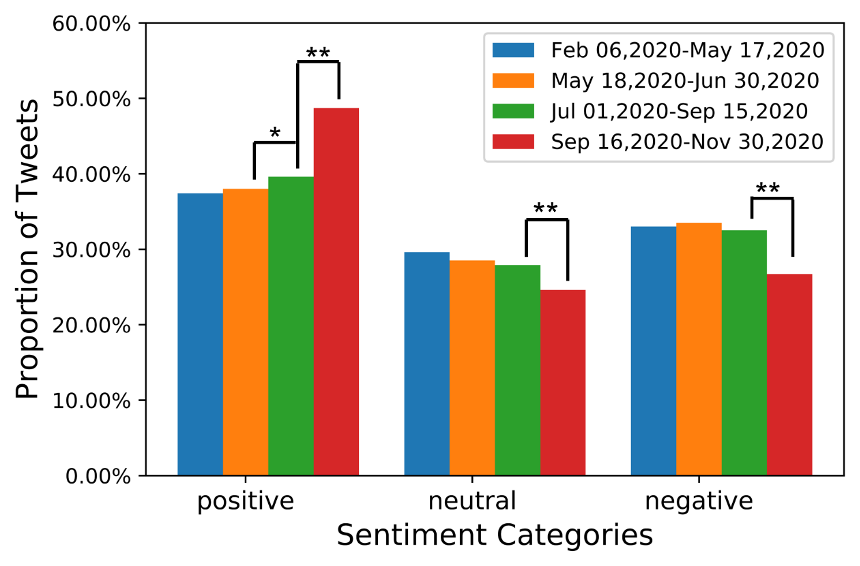

Supplement: Multimedia Appendix 1 [file publichealth_v8i7e34114_app1.png]

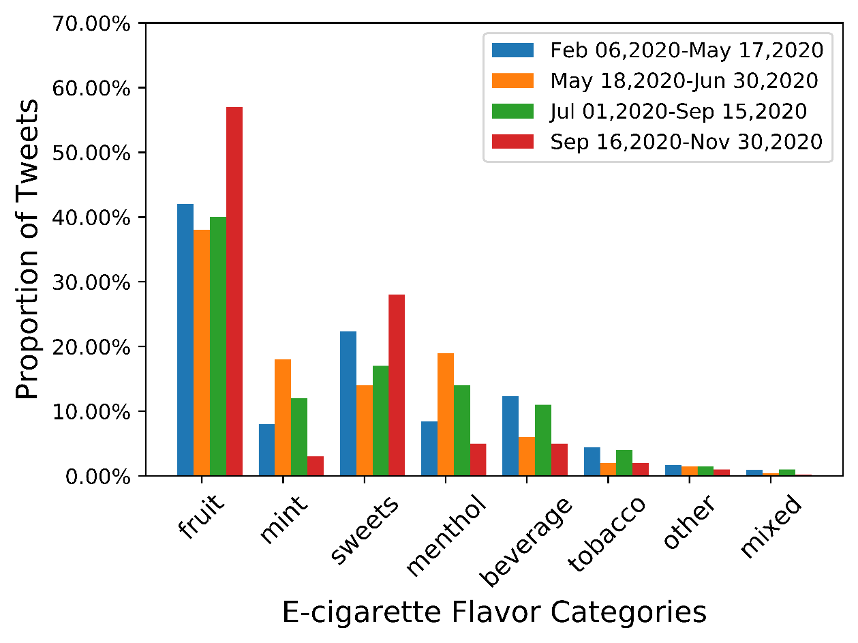

Supplement: Multimedia Appendix 2 [file publichealth_v8i7e34114_app2.png]
